# Transglutaminase 2 knockout mice are protected from bleomycin‐induced lung fibrosis with preserved lung function and reduced metabolic derangements

**DOI:** 10.14814/phy2.16012

**Published:** 2024-06-20

**Authors:** Margaret A. T. Freeberg, Thomas H. Thatcher, Sarah V. Camus, Linghong Huang, John Atkinson, Wade Narrow, Jeannie Haak, Andrew M. Dylag, L. Ashley Cowart, Timothy S. Johnson, Patricia J. Sime

**Affiliations:** ^1^ Division of Pulmonary Disease and Critical Care Medicine Virginia Commonwealth University Richmond Virginia USA; ^2^ Division of Pulmonary and Critical Care Medicine University of Rochester Rochester New York USA; ^3^ UCB Pharma SA Slough Berkshire UK; ^4^ Department of Pediatrics, Division of Neonatology University of Rochester Medical Center Rochester New York USA; ^5^ Department of Biochemistry and Molecular Biology Virginia Commonwealth University Richmond Virginia USA; ^6^ Present address: Mestag Therapeutics Cambridge UK; ^7^ Present address: Department of Surgery University of Rochester Rochester New York USA

**Keywords:** metabolism, metabolomics, pulmonary fibrosis, transglutaminase

## Abstract

Pulmonary fibrosis is an interstitial scarring disease of the lung characterized by poor prognosis and limited treatment options. Tissue transglutaminase 2 (TG2) is believed to promote lung fibrosis by crosslinking extracellular matrix components and activating latent TGFβ. This study assessed physiologic pulmonary function and metabolic alterations in the mouse bleomycin model with TG2 genetic deletion. TG2‐deficient mice demonstrated attenuated the fibrosis and preservation of lung function, with significant reduction in elastance and increases in compliance and inspiratory capacity compared to control mice treated with bleomycin. Bleomycin induced metabolic changes in the mouse lung that were consistent with increased aerobic glycolysis, including increased expression of lactate dehydrogenase A and increased production of lactate, as well as increased glutamine, glutamate, and aspartate. TG2‐deficient mice treated with bleomycin exhibited similar metabolic changes but with reduced magnitude. Our results demonstrate that TG2 is required for a typical fibrosis response to injury. In the absence of TG2, the fibrotic response is biochemically similar to wild‐type, but lesions are smaller and lung function is preserved. We also show for the first time that profibrotic pathways of tissue stiffening and metabolic reprogramming are interconnected, and that metabolic disruptions in fibrosis go beyond glycolysis.

## Graphical abstract

Tissue transglutaminase 2 (TG2) knockout mice demonstrated preserved lung function and reduced lesion area following bleomycin injury. Bleomycin fibrotic injury is associated with energy and amino acid metabolic changes. These metabolic changes are similar but overall reduced in magnitude in the TG2 KO mice. We also show for the first time that profibrotic pathways of tissue stiffening and metabolic reprogramming are interconnected, and that metabolic disruptions in fibrosis go beyond glycolysis.

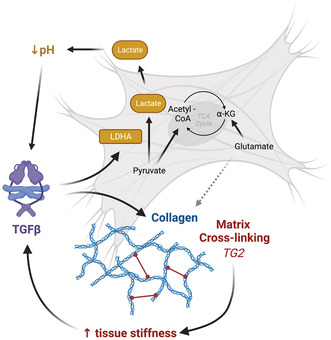

## 1 INTRODUCTION

Idiopathic pulmonary fibrosis (IPF) is a fatal progressive scarring disease of the lung. Lung fibrosis is characterized by interstitial thickening due to the excess deposition of extracellular matrix (ECM) and accumulation of mesenchymal cells, impairing gas exchange. The underlying etiology of IPF progression is incompletely understood. There are two current FDA‐approved therapies, pirfenidone and nintedanib, that slow the progression of disease in some patients, but additional therapies are urgently needed (King et al., 2014; Richeldi et al., 2014). An emerging paradigm is that fibrosis is driven by multiple processes with overlapping signals and effectors, limiting the efficacy of targeting single pathways. These compounding feed‐forward loops that contribute to the progression of disease include epithelial injury, dysregulated cellular metabolic metabolism, aging, senescence, and increased matrix production and stiffness altering the mechanical environment (Freeberg et al., 2021; Rackow et al., 2020).

Tissue transglutaminase 2 (TG2) is widely expressed and has multiple functions, including acting as an intracellular G‐protein, crosslinking extracellular proteins, facilitating fibronectin export from cells, and acting as a membrane‐associated anchor linking cells to ECM proteins including integrins (Belkin, 2011). TG2 catalyzes the formation of posttranslational bonds between glutamine and lysine residues as well as the deamidation of glutamine (Siegel & Khosla, 2007; Tabolacci et al., 2012). TG2 is reported to play a role in fibrosis in multiple organs, including kidney (Johnson et al., 2007), liver (Poole et al., 2019), heart (Wang et al., 2018), and lung (Olsen et al., 2011, 2014). TG2 is believed to contribute directly to fibrosis pathogenesis through crosslinking and stabilizing the ECM (Akimov & Belkin, 2015), and indirectly through activating latent TGFβ in the ECM, which facilitates fibroblasts to myofibroblast differentiation and activation of fibrotic functions of (myo)fibroblasts (Huang et al., 2010; Benn et al., 2019).

We and others have demonstrated that lactate is upregulated in IPF, which is significant because lactate in lung tissue can activate latent TGFβ, an important profibrotic cytokine, leading to a profibrotic feed‐forward loop (Kottmann et al., 2012; Xie et al., 2015). Additionally, glutamine metabolism has been demonstrated to be enhanced in fibrosis (Hamanaka et al., 2019; Roque & Romero, 2021; Wang et al., 2022). It is becoming evidently clear that lung fibroblasts respond to mechanical cues from their environment, and respond to increased matrix stiffness by expressing profibrotic phenotypes (Balestrini et al., 2012). We have previously reported the effect of TG2 genetic knockout in the mouse bleomycin fibrosis model (Olsen et al., 2011, 2014). He we are extending the study by considering lung physiology and function, tissue stiffness, and how that links to lactate as a driver of fibrosis. As TG2 crosslinking is one of the primary drivers of matrix stiffness in fibrosis (Steppan et al., 2017), we investigated whether TG2 deficiency alters lung tissue mechanical properties in the mouse bleomycin fibrosis model, and whether there is a link between TG2, matrix stiffness, and altered cellular metabolism.

## 2 MATERIALS AND METHODS

### 2.1 Mouse model of bleomycin‐induced lung fibrosis

All animal procedures were carried out at the University of Rochester under the supervision of the University Committee on Animal Research (UCAR) under protocol 2004‐335. Mice used for these experiments were male C57BL/6J purchased commercially (The Jackson Laboratories, Bar Harbor, Maine, USA) or TG2 knockout mice bred in‐house. The TG2 knockout mice are strain Tgm2^tm1.1Rmgr^ (Victor Chang Institute, New South Wales, Australia) in which exons 6–8 of the Tgm2 gene were removed by Cre‐mediated recombination (Nanda et al., 2001). Cre recombinase is from topoisomerase from bacteriophase P1 that catalyzes the site specific recombination of DNA between loxP sites. The strain was subsequently backcrossed at least 10 generations to C57BL/6J, which was verified by genome scanning. Adult male mice (age 8–12 weeks) received 2 U/kg bleomycin (Fresenius Kabi, Bad Homburg, Germany) in 40 μL saline by oropharyngeal aspiration (Lakatos et al., 2006) while under isoflurane anesthesia. After 21 days, the mice were euthanized by injecting a mixture of ketamine 100 mg/kg plus xylazine 20 mg/kg plus acepromazine 3 mg/kg, followed by exsanguination once the mice reached a surgical plane of anesthesia. The heart and lungs were removed *en bloc*. The right bronchus was tied off, and the right lung lobes were dissected and frozen individually in liquid nitrogen, then transferred to a −80°C freezer until analyzed. The left lung was inflated with 2% low melting point agarose and placed in 10% neutral buffered formalin, or was inflated with neutral buffered formalin and then placed in formalin overnight. A total of 15–17 mice per group were used, over two independent experiments (*N* = 5 and 10–12 mice per group, respectively). The frozen upper and lower right lobes were used for hydroxyproline assay and the middle right lobe was used for RNA extraction.

### 2.2 Histological analysis

Lung sections were stained with hematoxylin and eosin (H&E). For second harmonic generation (SHG) microscopy, FFPE sections were dewaxed, rehydrated, and scanned using a Genesis 200 system (HistoIndex Pte Ltd, Singapore). Collagen was revealed by SHG and the rest of the tissue reflected by two‐photon excitation fluorescence. Lung sections were scanned using a 20× objective with full laser power and 512 × 512 pixels resolution. Definiens software (Definiens AG, Germany) was used to quantify the SHG images. An algorithm was trained to detect large bundles of mature collagen around airways and blood vessels as a region of interest (ROI) identified as “airway collagen” which was excluded from further analysis. Non‐airway, or parenchymal, tissue was defined as a second ROI, identified as “tissue”. Within the tissue ROI, collagen and non‐collagen areas were quantified separately, and the results expressed as collagen area per total (collagen plus non‐collagen) areas, normalized to the average of the C57BL/6 saline group.

We obtained 10 serial sections from each block for immunohistochemistry, with two sections per slide (5 slides total). Slide 3 was stained for LDHA and isotype control (one section each). Slide 4 was stained with Gomori's trichrome (both sections). Slide 5 was stained with H&E (both sections). Lower power images (4× objective) from the H&E‐stained sections are directly contiguous with the other sections and higher power (10×) images were taken for the sections stained for trichrome, LDHA, and isotype controls to ensure localization continuity.

### 2.3 RNA quantification

RNA was purified from the right middle lung lobe using QIAzol extraction with tissue pulverization with a bead blender, followed by purification with RNAeasy kit (Qiagen, Germany, cat. no. 74104) according to the manufacturer protocols. Reverse transcription to cDNA was completed with iScript Supermix (Bio‐Rad, USA, cat. no. 1708840). Real‐time PCR was completed with 1 ng of sample cDNA using Bio‐Rad pre‐validated primers: Col3a1, qMmuCED0006332; Col1a1, qMmuCED0044222; Fn1, qMmuCED0045687; Gapdh, qMmuCED0027497.

### 2.4 Lung function testing

For mouse lung function testing, the mice were anesthetized with ketamine/xylazine/acepromazine as described above, then the trachea was cannulated, and the mice were connected to a ventilator attached to a flexiVent instrument (SciReq). The mice were paralyzed with 2 mg/kg vecuronium bromide intraperitoneally to prevent spontaneous breathing effort, and the flexiVent performed a series of forced ventilation maneuvers that were used to derive lung function parameters. Then the mice were euthanized for tissue harvest as described.

### 2.5 Western blotting

Total lung tissue was homogenized in water, and protein was extracted in 1X RIPA buffer with protease inhibitors. Equal amounts of protein were analyzed by western blot using antibodies to LDHA (Cell Signaling, 2012S, 1:1000 dilution) and beta‐tubulin (Abcam, ab6046, 1:10,000 dilution), followed by secondary antibodies (Goat anti‐Rabbit, Jackson Immuno, Cat# 1111‐035‐144) conjugated to HRP, and detected by chemi‐luminescence (Millipore Sigma, Cat# WBKLS0500) on a C‐digit scanner (Li‐Cor, Lincoln, NE).

### 2.6 Lactate assay

Lactate in tissue homogenates was determined by L‐Lactate Assay Kit (Abcam, #AB65330) according to the manufacturer's protocol.

### 2.7 TGFβ assay

TGFβ in lung tissue homogenates was determined using HEK293FT cells stably transfected with a TGFβ‐dependent luciferase reporter as previously described (Woeller et al., 2015).

### 2.8 Untargeted metabolomics

Lung homogenates were analyzed at the VCU Lipidomics and Metabolomics Shared Resource. Methanol was added to each sample in a 1:1 ratio to precipitate proteins. Samples were cleared by centrifugation at 5000 rpm for 5′ and subjected to liquid chromatography/tandem mass spectrometry using the Q Exactive Orbitrap instrument (Thermo Fisher Scientific, Bremen, Germany). Data were collected and features were identified using Compound Discoverer 3.1 (m/z cloud and ChemSpider). We identified 45 significantly changing named compounds compared to saline‐treated wild‐type mice at an uncorrected *p*‐value ≤0.05. The raw data have been deposited online with *DOI pending*. Pathway enrichment was performed using small molecule pathway database (SMPDB) and KEGG Pathway enrichment in MetaboAnalyst.

### 2.9 Statistical analysis

Results were analyzed using a two‐way ANOVA with Sidak's multiple comparisons test.

## 3 RESULTS

### 3.1 TG2 deficiency attenuates fibrosis and expression of fibrosis‐related genes

**FIGURE 1:**
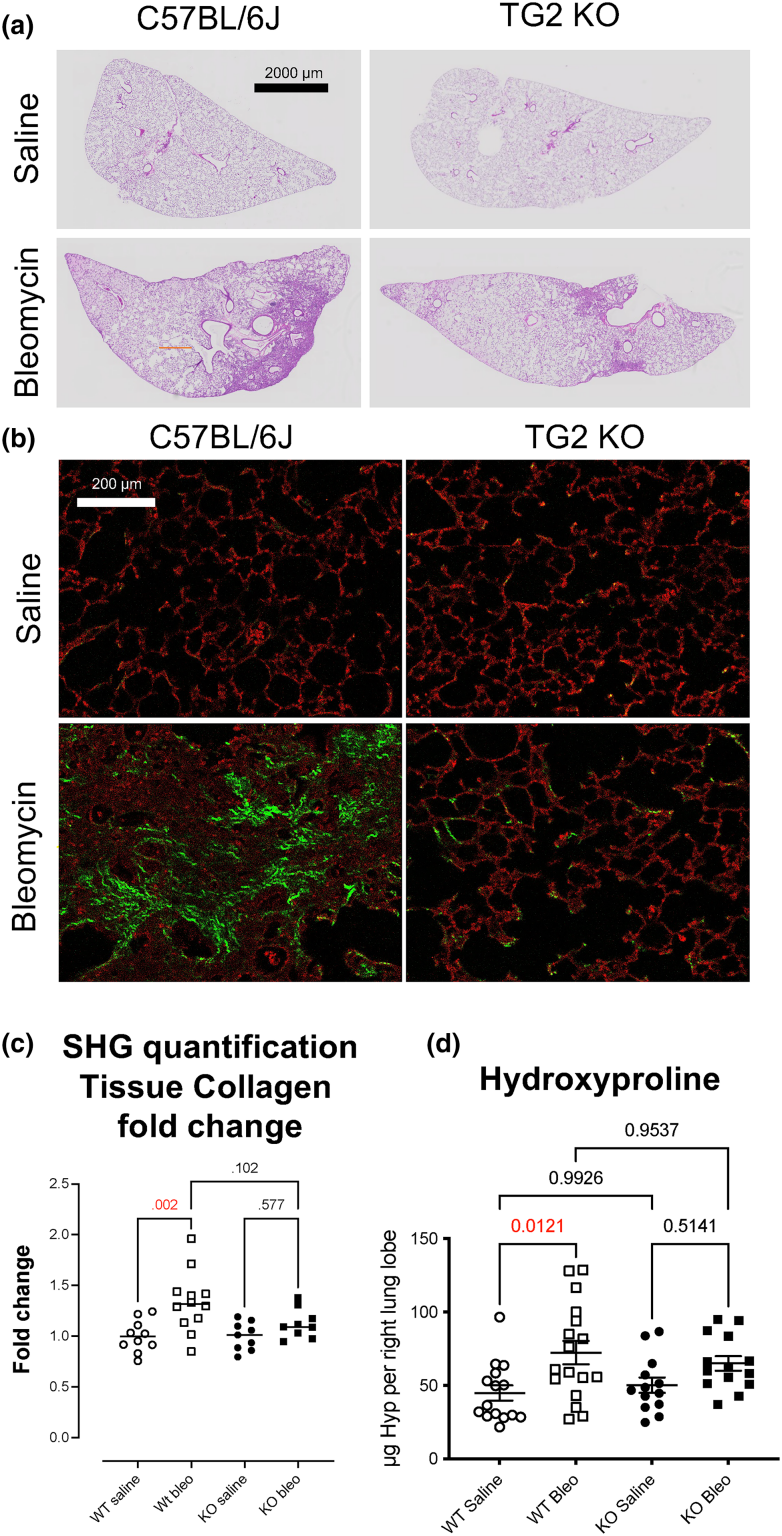
TG2 deficiency attenuates lung fibrosis in the bleomycin mouse model. C57BL/6 and TG2 KO mice were treated with 2 units/kg bleomycin and harvested on Day 21 as described. Lung tissue was inflated and fixed, and FFPE sections were prepared. (a) Representative whole slide images of H&E‐stained sections. (b) Collagen was detected in FFPE sections using SHG microscopy. Green pixels indicate collagen, red pixels indicate non‐collagen tissue, and black pixels indicate air spaces (airways, alveoli, or blood vessels). Representative regions are shown. White arrows highlight collagen present around airways. Orange boxes highlight collagen changes in the parenchyma. (c) SHG collagen was quantified removing airway collagen and expressed as fold change normalized to saline‐treated C57BL/6 mice. To quantify collagen in parenchymal tissue, an algorithm was used to exclude collagen around large airways and blood vessels, and collagen in the parenchyma (green pixels) was determined as a percentage of total tissue (red + green pixels). *N* = 9–13 mice per group. (d) Collagen as detected by hydroxyproline in total lung tissue *N* = 15 mice per group. *p*‐values from two‐way ANOVA with Sidak's post‐test.

C57BL/6 (wild‐type) and TG2 KO male mice were treated with bleomycin (2 units/kg) and harvested for analysis of fibrosis. One effect of bleomycin administration is to cause transient weight loss in mice; here, the TG2 KO mice lost less weight than wild‐type, although the effect was not significant (Figure S1). Tissue sections from the left lung show that bleomycin‐induced fibrosis was reduced in the KO mice (Figure 1a). Collagen in FFPE tissue sections was imaged and quantified with SHG microscopy (Figure 1b). Collagen is indicated by green pixels and other tissues by red pixels. Collagen is present around airways and blood vessels in untreated as well as bleomycin‐treated lungs (Figure 1b, white arrows). Collagen in the parenchyma is strongly induce by bleomycin in the wild‐type but not the TG2 KO mice. To quantify collagen in parenchymal tissue, an algorithm was used to exclude collagen around large airways and blood vessels, and collagen in the parenchyma (green pixels) was determined as a percentage of total tissue (red + green pixels). Results are expressed as fold change over wild‐type mice treated with saline. Wild‐type mice exhibited a statistically significant 34% increase in parenchymal collagen after bleomycin, while the TG2 KO mice exhibited a 12% increase in parenchymal collagen that was not significant compared to saline controls (Figure 1c). Similarly, hydroxyproline assay identified a 60% increase in lung tissue hydroxyproline in the wild‐type but not in the TG2 KO mice (Figure 1d).

**FIGURE 2:**
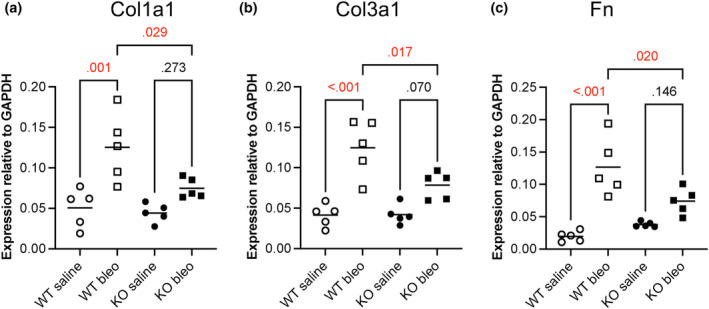
TG2 deficiency attenuates the expression of fibrosis‐related genes. C57BL/6 and TG2 KO mice were treated with bleomycin and harvested as described above. RNA was extracted from the right middle lung lobe, and expression of (a) collagen 1, (b) collagen 3, and (c) fibronectin determined by RT‐PCR and normalized to GAPDH. *N* = 5 mice per group. *p*‐values are by one‐way ANOVA with Tukey's post‐test for multiple comparisons.

We next analyzed the expression of key matrix genes collagen and fibronectin. Collagen gene expression was markedly reduced in TG2 KO mice treated with bleomycin compared to wild‐type mice. Bleomycin induced collagen expression 2.5‐fold (Col1A1) and threefold (Col3A1) in C57BL/6 mice but less than twofold in the TG2 KO mice. Further, the increased collagen expression in TG2 KO mice was not statistically significant (Figure 2a,b). A similar result was observed for fibronectin, where bleomycin upregulated fibronectin expression in the C57BL/6 but not in the TG2 KO mice (Figure 2c). Finally, TG2 expression was upregulated 2.2‐fold in C57BL/6 mice but was not detected in TG2 KO mice, as expected (Figure S2).

### 3.2 TG2 deficiency preserves lung function after bleomycin

**FIGURE 3:**
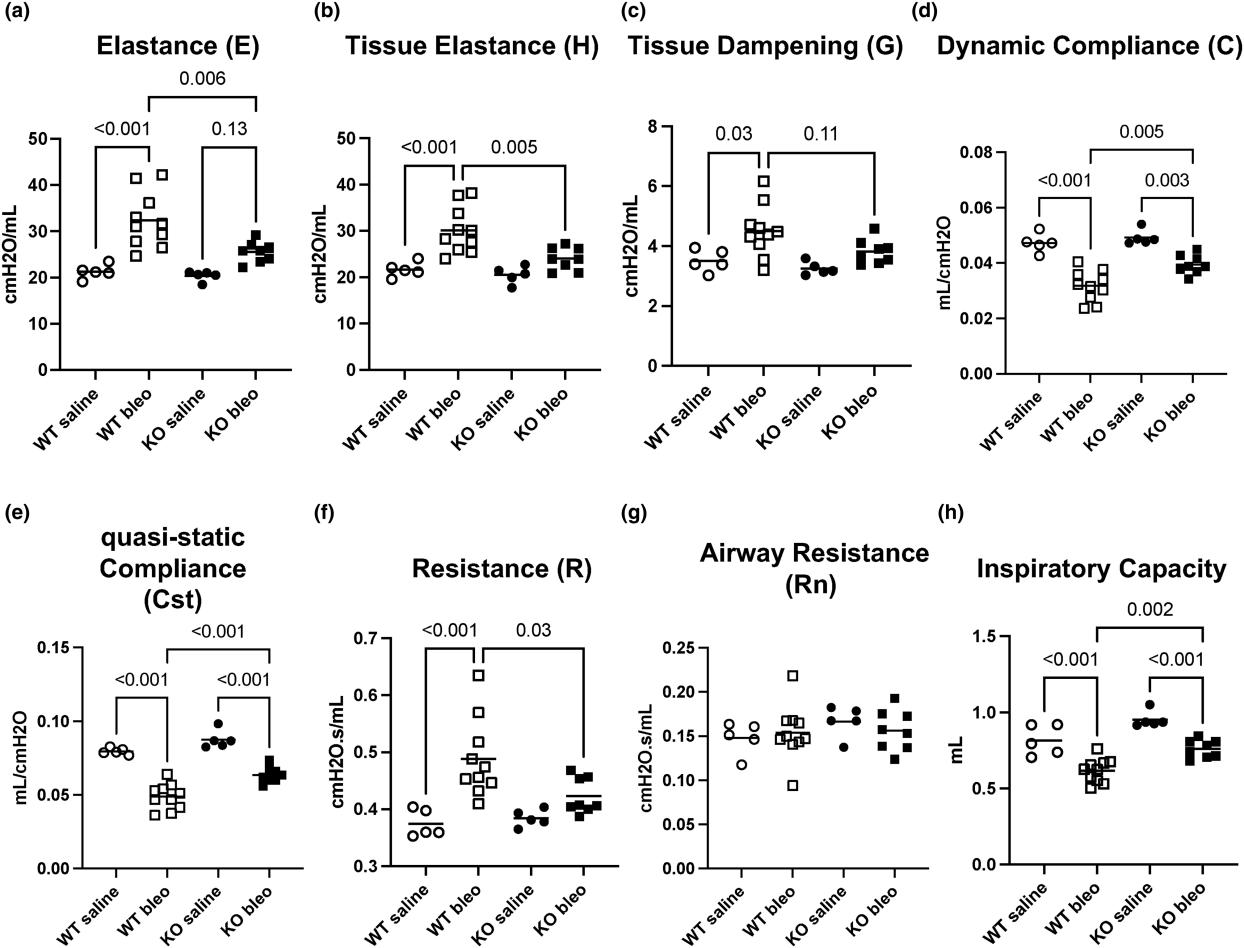
TG2 deficiency preserves lung function in bleomycin‐treated mice. C57BL/6 and TG2 KO mice were treated with bleomycin as described above. Immediately prior to euthanasia (Day 21), lung function was measured with a flexiVent ventilator. (a) Elastance, (b) Tissue elastance (*H*), (c) Tissue dampening (*G*), (d) dynamic compliance (*C*), (e) Quasi‐static compliance, (f) Resistance (*R*), (g) airway resistance (RN), and (h) Inspiratory capacity from PV loop Area. PV, pressure–volume. *N* = 9–13 mice per group. *p*‐values from two‐way ANOVA with Sidak's post‐test.

Prior to tissue harvest, we measured pulmonary function in the mice with a flexiVent apparatus. This instrument uses a series of forced ventilator movements to derive information about the mouse lung structure and function like airways resistance and compliance, similar to human pulmonary function testing. Results are shown in Figure 3. Functional testing showed that bleomycin caused significant changes to several lung function parameters, while TG2 deletion led to remarkable preservation of lung function. Elastance (*E*) is the elastic stiffness of the total respiratory system and includes contributions from the tissue, chest wall, and airways, while tissue elastance (*H*) represents the elastance of the lung parenchyma. Relative to saline‐treated mice, bleomycin significantly increased the elastances in the wild‐type mice but not in the TG2 KO mice (Figure 3a,b). Tissue dampening (*G*), reflecting energy dissipation in the alveoli, is significantly increased in wild‐type mice injured with bleomycin compared to saline but is unchanged in TG2 KO mice (Figure 3c). Tissue elastance (*H*) and damping (*G*) are sensitive to viscoelastic mechanical changes present in the lung. Dynamic compliance (*C*) is a measure of the ease with which the respiratory system can be extended; it is the inverse of elastance (*E*) (Figure 3d). Static compliance is normally measured during a breath‐holding maneuver; here it is reported as quasi‐static compliance (Cst) since it is measured during a single deep inflation by the ventilator. Bleomycin significantly reduces compliance in both wild‐type and TG2 KO mice, although the effect in TG2 KO mice is of a significantly smaller magnitude (Figure 3e).

Using the flexiVent system, R is the total resistance of the respiratory system (including airways, parenchyma, and chest wall) while Rn, or Newtonian resistance, is an estimate of airways resistance derived from the forced oscillation technique. Since the chest wall of mice is extremely flexible, it is assumed to not contribute to total resistance (*R*). Therefore, if *R* changes but Rn does not change, the change in *R* must be due to parenchymal resistance. Here, total resistance (*R*) is significantly increased in the C57BL/6 mice treated with bleomycin but not in the TG2 KO mice (Figure 3f), and there was significant preservation of resistance in TG2 KO mice with bleomycin relative to C57BL/6 mice plus bleomycin. Rn was not affected by bleomycin in either group (Figure 3g), which is consistent with bleomycin causing fibrosis in the parenchyma but not airways in this model. The inspiratory capacity is calculated from the pressure‐volume loop area. Wild‐type mice exhibited a significantly larger reduction in inspiratory capacity (volume), after bleomycin compared to TG2 KO mice (Figure 3h). Taken together, these results show a remarkable preservation of lung function in the TG2 KO mice following bleomycin administration.

### 3.3 Reduced metabolic reprogramming in TG2 deficient mice

**FIGURE 4:**
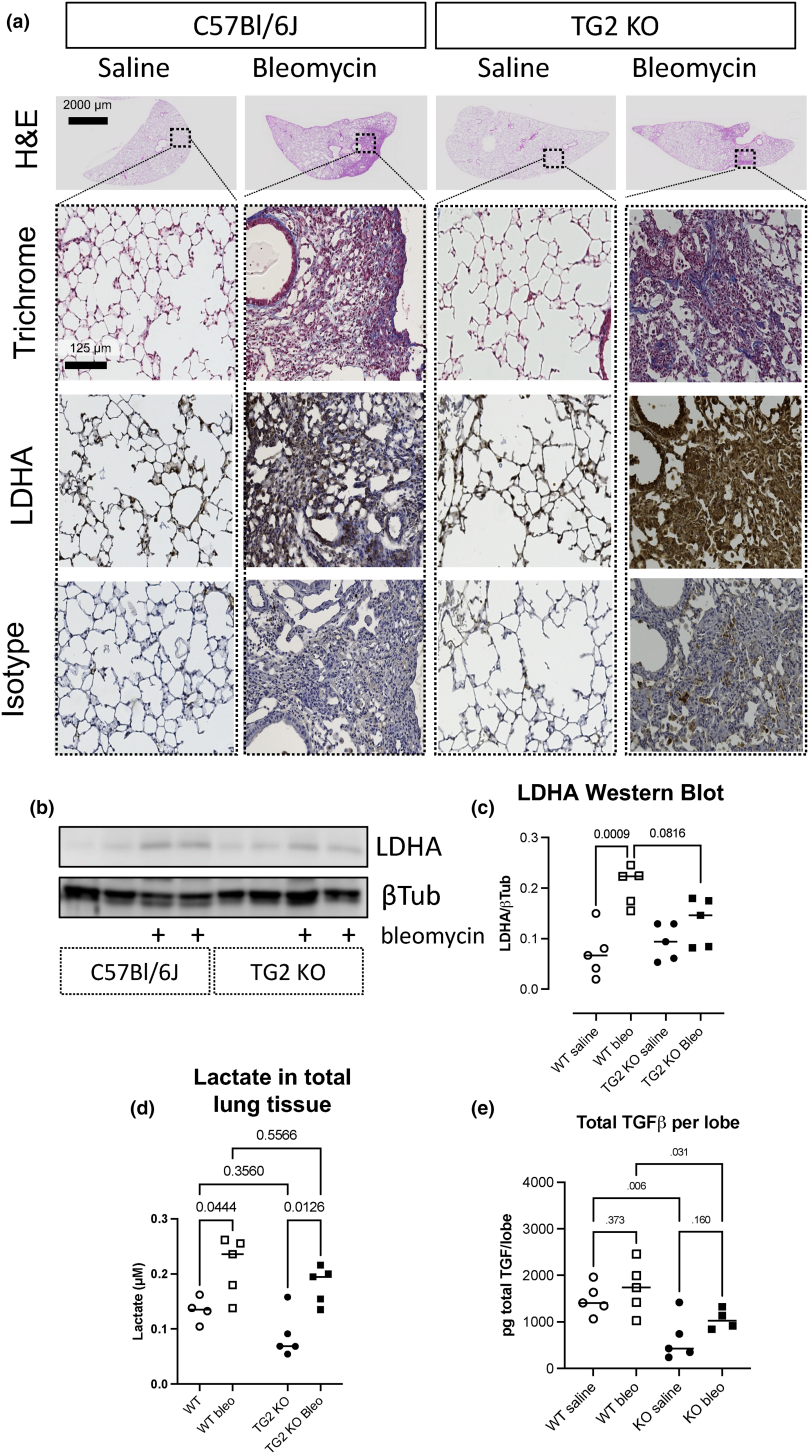
Lactate glycolytic reprogramming is reduced in TG2 deficient mice. (a) Serial sections were stained with Gomori's trichrome, or immunostained for LDHA, and isotype control. Control sections are in normal parenchymal area. Bleomycin sections are from fibrotic lesions. H&E images were taken with a 4× objective and Gomori's trichrome, LDHA, and isotype control images were taken with a 10× objective (4× scale bar = 500 μm, 10× scale bar = 100 μm). The dashed box in the 4× H&E images shows the regions that were photographed at 10× for the other stains. (b) Western blot of total lung tissue homogenates for LDHA normalized to loading control beta‐tubulin. (c) Quantification of densitometry for LDHA/βTubulin; *n* = 4–5 per group. (d) Lactate in total lung tissue homogenates. (e) Total TGFβ per upper right lung lobe.

We have previously reported that IPF lung tissue exhibits increased glycolysis, including increased expression of lactate dehydrogenase (LDH) A and production of lactate, and that lactate promotes fibrogenesis in vitro by activating latent TGFβ, which promotes myofibroblast differentiation (Kottmann et al., 2012). We wanted to examine whether there was a link between lung tissue metabolic changes and matrix and tissue stiffness changes in pulmonary fibrosis by interrogating LDHA and other metabolic pathways in the TG2 KO mice. Here we report that LDHA is upregulated by bleomycin in wild‐type mice, but upregulation is attenuated in TG2 deficient mice. Total LDHA expression in lung tissue was reduced in TG2 KO mice treated with bleomycin compared to wild‐type bleomycin‐treated mice, although LDHA expression within fibrotic lesions (as detected by immunohistochemistry) was similar in the two strains (Figure 4a–c). We also detected a trend of reduced lactate in tissue homogenates of TG2 KO mice, but this was not significant (Figure 4d). We observed a significant reduction in total TGFβ in the lungs of TG2 deficient mice, consistent with these mice developing less fibrosis (Figure 4e).

To investigate the effect of bleomycin and TG2 deficiency on additional cellular metabolic pathways, we performed untargeted metabolomics on total lung tissue homogenates.

**FIGURE 5:**
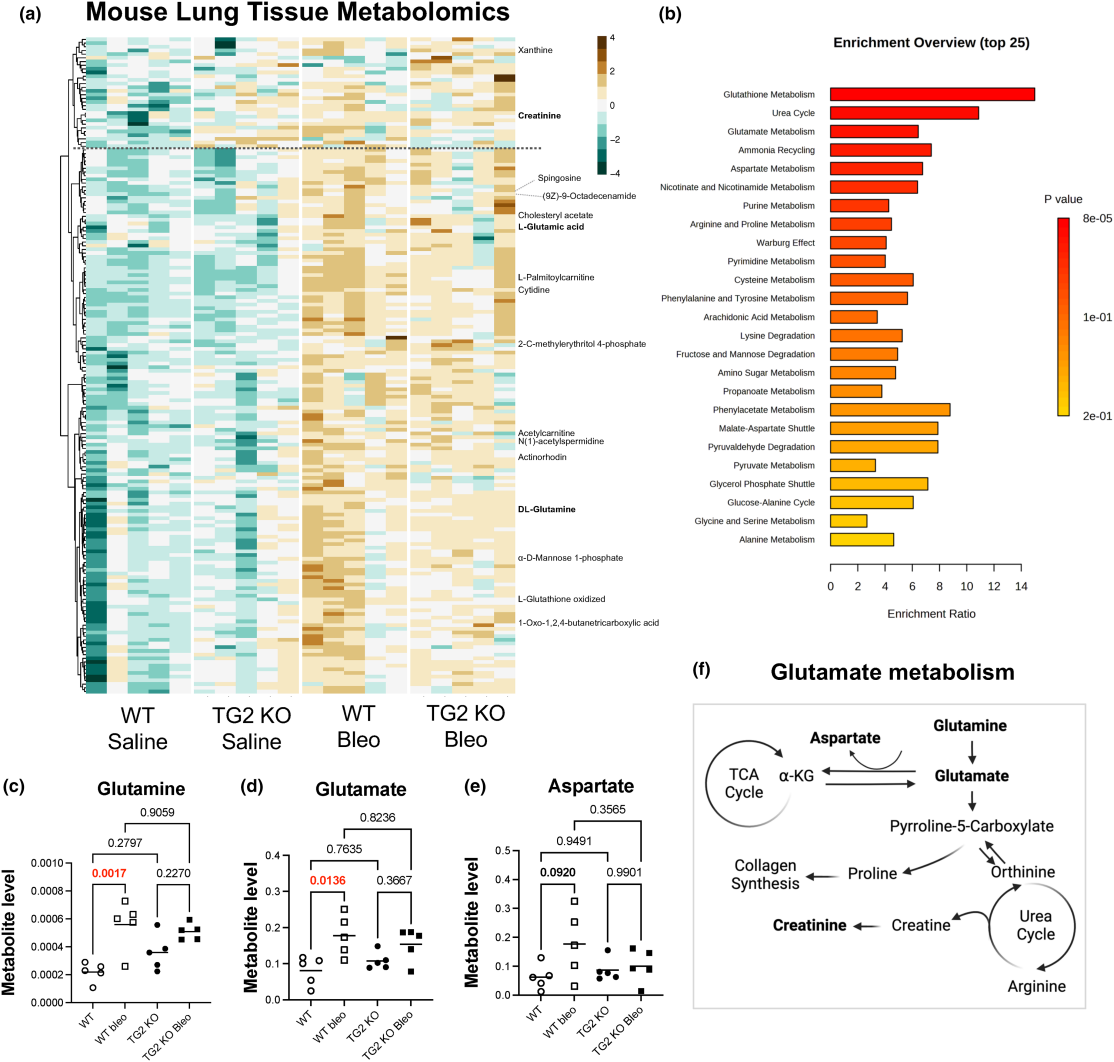
Untargeted metabolomics reveals altered amino acid and energy metabolism. (a) Heatmap of significantly changing features (uncorrected *p* ≤ 0.05, log2 fold change>|1|). One hundred seventy‐eight total spectral features. Seventeen named compounds. (b) MetaboAnalyst pathway enrichment of known metabolites identified in the dataset. (c–e) Key metabolites in the glutamine pathway are shown, normalized to total detections. Points represent individual mice, *p*‐values are from two‐way ANOVA with Sidak's post‐test. (f) Glutamate metabolism can provide alternative energy inputs into the TCA cycle or resources for collagen synthesis.

One hundred seventy‐nine unique spectral features were identified as having at least a twofold change with a *p* < 0.05 versus WT or TG2 saline. The 179 spectral features are shown in the heatmap in Figure 5a. Of the 179 features, 17 of them were identifiable with high confidence, as annotated on the heatmap (Figure 5a). SMPDB pathway enrichment was performed in MetaboAnalyst. Pathway analysis reveals that mice with bleomycin‐induced fibrosis exhibit alterations in pathways related to the glutamine cycle, proline metabolism, and the TCA cycle (Figure 5b). Some key metabolites of interest are highlighted (Figure 5c–e).

Glutathione metabolism is enriched due to the detection of oxidized glutathione (GSSG) in the metabolite list. We highlight the next two highest enrichment pathways, urea cycle, and glutamate metabolism. Of interest, fibrotic lungs contain increased levels of glutamine, glutamate, and aspartate, which are not significantly increased with TG2 deficiency (Figure 5c–e). We observe hints of both pathways being present in the WT mouse with significant increases in glutamine, the glutamate precursor, glutamate, and a trended increase in aspartate. All of these metabolites fail to increase in the TG2 KO mice following bleomycin challenge. Taken together, these data highlight the interconnected nature of profibrotic feed‐forward loops. Here we highlight how the genetic deletion of TG2 has overall impacts on the development of fibrosis including reducing energy and glutamate metabolism changes typically present in the fibrotic lung.

## 4 DISCUSSION

The overall objective of this study was to evaluate the role of TG2 in a mouse model of pulmonary fibrosis and determine whether there is a connection between tissue stiffness and altered cellular energy metabolism. Tg2 has several known functions that contribute to fibrosis progression (Benn et al., 2019; Soltani & Kaartinen, 2023). TG2 is primarily thought to play a role in fibrosis due to its role in matrix stabilization and crosslinking due to its catalytic activity; however, there are several other roles that contribute to fibrosis pathogenesis. Disruptions in any one of these pathways could disrupt the development of fibrosis. We hypothesized that TG2 deficiency would inhibit bleomycin‐induced fibrosis by two routes–reducing incorporation and activation of latent TGFβ in the ECM and reducing lung tissue stiffness and stiffness‐driven metabolic changes.

Consistent with our previous report (Olsen et al., 2011), TG2‐deficient mice were protected from fibrosis, not only with smaller lesions, reduced tissue collagen, and reduced expression of profibrotic matrix genes but also with significantly preserved lung function. Wild‐type mice treated with bleomycin exhibited increased elastic stiffness (elastance), decreased compliance, and decreased inspiratory capacity, all of which are consistent with physiological changes in human cases of IPF and other fibrosing ILDs (Plantier et al., 2018). Although we did not directly measure lung tissue stiffness in these mice, elastance and compliance are good indirect indicators of lung tissue stiffness, and our results support the hypothesis that ECM crosslinking and tissue stiffness are reduced in the TG2 KO mice. Forced vital capacity (FVC) is an important clinical measure for evaluating patients with pulmonary fibrosis and has been used as an endpoint in some clinical trials. The flexiVent system can estimate FVC but we did not include those parameters in our study. We acknowledge that it will be important to include FVC in future studies to help connect mouse model results to clinical endpoints.

Since the expression of both TG2 and LDHA is regulated by TGFβ, TGFβ may represent the intersection point of these two pathways. Alternatively, it was recently reported that TG2 deletion in breast cancer cells reduced LDHA expression, and TG2 overexpression increased LDHA expression, via the MEK/ERK pathway (Xu et al., 2022). In either case, these profibrotic pathways are interconnected, and TG2 may represent an interventional target for multiple profibrotic pathways. TG2 crosslinking of latent TGFβ‐binding proteins plays a role in TGFβ activation (Lockhart‐Cairns et al., 2022). Here we suspect the decrease in total TGFβ is due to failed storage and linking of TGF‐β to TG2 in the matrix. This result is consistent with results investigating TG2 in kidney fibrosis (Johnson et al., 2007).

We have previously reported that TGFβ upregulates LDHA expression and lactate export in myofibroblasts, leading to decreased extracellular pH and activation of latent TGFβ, creating a profibrotic feed‐forward loop (Kottmann et al., 2012, 2015). Compared to wild‐type mice treated with bleomycin, TG2 deficiency resulted in decreased expression of LDHA. However, there was no effect on production of lactate (Figure 4d). It is important to recognize that there are multiple potential metabolic sources of lactate. Here we have examined the role of traditional glycolytic metabolism where lactate is produced from pyruvate with LDH. Lactate can also be produced from malate with malate–lactate transhydrogenase and from the methylglyoxal pathway with glyoxalase I and II or aldehyde dehydrogenase. Additionally, there are multiple sources of pyruvate, therefore lactate will be produced as long as LDHA is present, and pyruvate is provided. Our recent findings in radiation‐induced lung fibrosis patients identify alternative sources of pyruvate and TCA cycle inputs as possible derangements present in lung fibrosis (Odoom et al., 2023). Ongoing work is investigating the primary source of lactate and pyruvate dependent on the profibrotic stimuli and to better understand the interaction between mechanics and metabolism.

We wanted to explore whether bleomycin fibrosis was associated with changes to other metabolic pathways beyond lactate using untargeted metabolomics (Figure 4). One limitation of untargeted approaches is that the mass spec returns features consisting of an exact mass and a retention time. There are many compounds with the same formula but different structures, requiring additional work is needed to correlate retention times with structures with sufficient confidence to label the feature as a known compound. This limits our pathway enrichment analysis. Furthermore, metabolomics approaches provide a snapshot of metabolite levels at a given moment. If a metabolite is changing (up or down) compared to saline controls, it indicates an alteration in reaction kinetics due to the production or consumption of a specific metabolite; enzyme activity, metabolite flux, and metabolite tracing are required to validate findings. Here we highlight changes to glutamate metabolism. Increased detection of glutamate could be a result of altered rate limiting enzyme activity, such as enhanced enzyme activity of 5‐oxoprolinase and/or reduced activity of enzymes responsible for the conversion of glutamate to downstream products, such as aspartate aminotransferase (GOT2). Biologically, increased glutamate and aspartate in bleomycin could be associated with increased energy input into the TCA cycle via α‐ketoglutarate to support the increased energy demands of fibrotic cells, or with increased collagen production, or both (Figure 5f). Previous reports demonstrated that glutamine metabolism is required for collagen synthesis in lung fibroblasts, and that the preferential fate of glutamine is toward collagen synthesis and not the TCA cycle in an in vitro model (Hamanaka et al., 2019). We recently reported similar alterations in the glutamate pathway in patients with radiation‐induced pulmonary fibrosis (Odoom et al., 2023). In accord with the lactate and LDHA results, TG2 KO mice exhibited similar metabolic changes to wild‐type mice after bleomycin treatment, although the magnitude of the changes was reduced. It is worth noting that there is a small cluster of features that are altered due to the TG2 genetic deletion. The named compounds within the cluster provide little mechanistic insight into pathways that are altered, but the presence of this cluster highlights the importance of understanding how TG2 deletion alone might alter the metabolome of the lung tissue.

In summary, we demonstrate that TG2 deletion results in reduced lung fibrosis with reduced tissue stiffness and preserved lung function. Bleomycin‐induced pulmonary fibrosis is marked by widespread metabolic changes suggestive of energy metabolism compensation of alternative energy inputs into the TCA cycle due to the excess production of lactate. We find that TG2 KO bleomycin fibrotic metabolism is qualitatively similar but quantitatively different, with reductions in key markers associated with fibrosis in the wild‐type mice. Taken together, this demonstrates that tissue stiffness and metabolism as linked, and that reducing tissue stiffness and reducing the size and propagation of the fibrotic lesion leads to reduced metabolic changes.

## AUTHOR CONTRIBUTIONS

The study was conceived and designed by THT, MATF, TSJ, and PJS. Experiments were performed and data were collected by THT, MATF, LH, JA, JH, AMD, WN, and SVC. Data were interpreted and analyzed by THT, MATF, SVC, LH, JA, LAC, TSJ, and PJS. The manuscript was written by THT and MATF. All authors read and approved the final draft.

## FUNDING INFORMATION

This research was supported in part by UCB Pharma SA. This research was supported in part by R03HL095402 and R01HL127001. MATF was supported by F32HL154525 and a Pulmonary Fibrosis Foundation Scholars grant. AMD was supported by a grant from the Strong Children's Research Center at the University of Rochester and an American Lung Association Catalyst Award (Award 699366). Services in support of the research project were provided by the VCU Massey Cancer Center Lipidomics and Metabolomics Shared Resource, supported, in part, with funding from NIH‐NCI Cancer Center Support Grant P30 CA016059.

## CONFLICT OF INTEREST STATEMENT

At the time these experiments were performed, LH, JA, and TSJ were employed by UCB Pharma SA. PJS has received consultancy fees from UCB Pharma SA and from other commercial entities with interests in pulmonary fibrosis. The other authors have no financial conflicts of interest.

## ETHICS STATEMENT

All animal procedures were ethically approved by the University Committee on Animal Research (UCAR) at the University of Rochester (protocol 2004‐335).

## Supporting information


Figure S1.
Figure S2.
