# Supplementary material for: Transglutaminase 2 knockout mice are protected from bleomycin‐induced lung fibrosis with preserved lung function and reduced metabolic derangements
Source: Physiol Rep. 2024 Jun 20;12(12):e16012. doi: 10.14814/phy2.16012 (PMC11189770; doi:10.14814/phy2.16012)
Supplement: Supplementary file 1 — Figure S1. Figure S2. [file PHY2-12-e16012-s001.pdf]

Figure S1

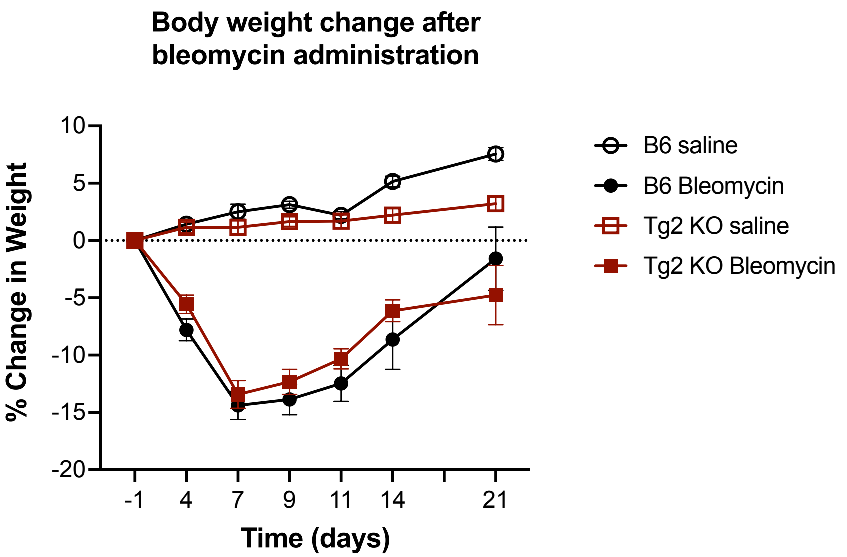

Figure S1. Bleomycin administration causes transient weight loss in mice. C57BL/6 (B6) and TG2 KO mice were treated with bleomycin as described. Mice were weighed every 2-3 days. The percent change from baseline (weight on day -1) is shown. The differences between B6 and KO mice are not significant.

Figure S2

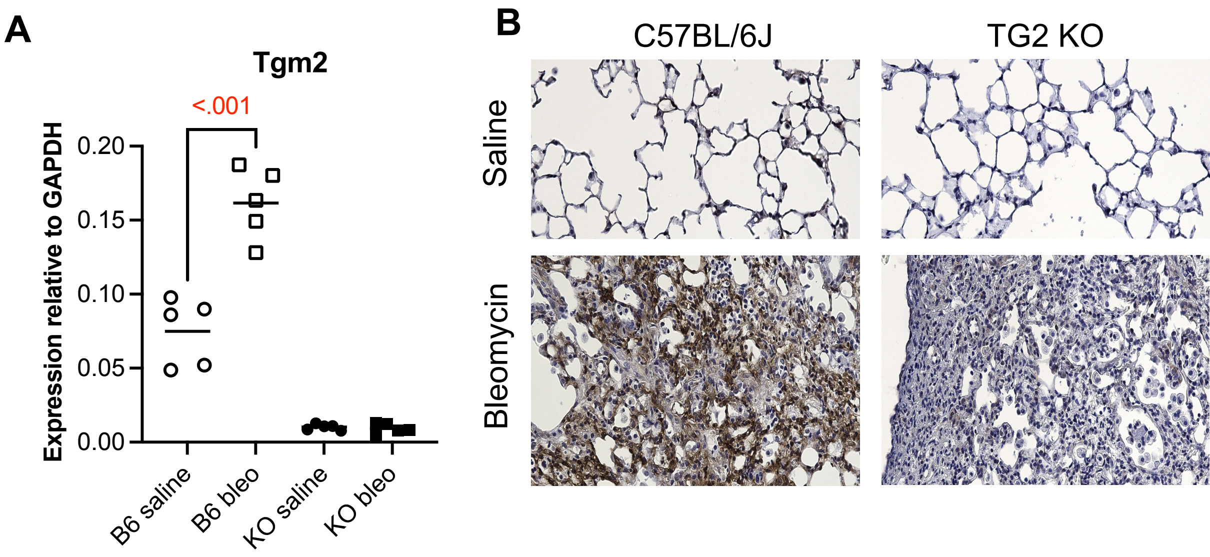

Figure S2. Confirmation of TG2 deletion. C57BL/6 (B6) and TG2 KO mice were treated with bleomycin as described. **A.** Tgm2 gene expression by RT-PCR (n=5). **B.** TG2 protein expression by immunohistochemistry.
